# Cognitive Performance Enhancement Induced by Caffeine, Carbohydrate and Guarana Mouth Rinsing during Submaximal Exercise

**DOI:** 10.3390/nu9060589

**Published:** 2017-06-09

**Authors:** Laura Pomportes, Jeanick Brisswalter, Laurence Casini, Arnaud Hays, Karen Davranche

**Affiliations:** 1 Laboratoire Motricité Humaine Expertise Sport Santé, Université Nice Sophia Antipolis, 06205 Nice, France; laurapomportes@hotmail.fr (L.P.); brisswalter@unice.fr (J.B.); 2 CREPS PACA, 13080 Aix-en-Provence, France; 3 Laboratoire de Neurosciences Cognitives, Aix-Marseille Université, CNRS, LNC, 13331 Marseille, France; laurence.casini@univ-amu.fr; 4 Institut des Sciences du Mouvement, Aix-Marseille Université, UMR 7287, 13288 Marseille, France; arnaud.hays@gmail.com; 5 Laboratoire de Psychologie Cognitive, Aix-Marseille Université, CNRS, LPC, 13331 Marseille, France

**Keywords:** nutrition, cognition, perceived exertion, mouth rinse, time-perception task, conflict task

## Abstract

The aim of this study was to investigate the influence of serial mouth rinsing (MR) with nutritional supplements on cognitive performance (i.e., cognitive control and time perception) during a 40-min submaximal exercise. Twenty-four participants completed 4 counterbalanced experimental sessions, during which they performed MR with either placebo (PL), carbohydrate (CHO: 1.6 g/25 mL), guarana complex (GUAc: 0.4 g/25 mL) or caffeine (CAF: 67 mg/25 mL) before and twice during exercise. The present study provided some important new insights regarding the specific changes in cognitive performance induced by nutritional supplements. The main results were: (1) CHO, CAF and GUA MR likely led participants to improve temporal performance; (2) CAF MR likely improved cognitive control; and (3) CHO MR led to a likely decrease in subjective perception of effort at the end of the exercise compared to PL, GUA and CAF. Moreover, results have shown that performing 40-min submaximal exercise enhances information processing in terms of both speed and accuracy, improves temporal performance and does not alter cognitive control. The present study opens up new perspectives regarding the use of MR to optimize cognitive performance during physical exercise.

## 1. Introduction

Several nutritional supplements are known to possess ergogenic effects on endurance performance (for review, see Reference [1,2]). The most largely studied are carbohydrate (CHO) and caffeine (CAF) assumed to help in limiting fatigue [3,4,5,6]. Both supplements have been shown to improve endurance capacity (exercise ≥ 2 h) but most likely utilizing two different mechanisms of action. After ingestion, CAF, which is thought to act as a central stimulant, is rapidly distributed to all tissues and readily crosses the blood-brain barrier to exert its ergogenic effects on the central nervous system mediated by the antagonism of adenosine receptors, which induces higher dopamine concentrations in the brain [7]. For CHO ingestion, the mechanism behind the ergogenic effect is rather a metabolic response, which would allow for maintaining plasma glucose concentration and high rates of CHO oxidation [8]. However, some studies have also shown that CHO supplementation can improve performance for high intensity exercises (>75% of maximal oxygen uptake (VO_2max_)) lasting 1 h or less [9,10]. These effects are difficult to explain by a metabolic hypothesis since CHO availability is not a limiting factor during the first hour of exercise [10]. Furthermore, Carter et al. [9] found that rinsing the mouth with a non-sweet maltodextrin solution significantly reduced the time to complete a 1-h cycle time trial. For the authors, the benefit observed could be the result of a central effect leading to the improvement of the motor drive or motivation, possibly due to the activation of receptors inside the mouth.

These ergogenic effects observed after mouth rinsing (MR) with nutritional supplements open up new perspectives in terms of performance optimization strategies. The fact that similar magnitude benefits have been observed on performance using MR or ingestion [11] incites to investigate thoroughly to define potential benefits and gainful usage, for example with athletes who are inclined to gastro-intestinal distress or when they need to limit energy intake (weight control). Studies using CHO MR [11,12,13,14,15,16] have generally reported exercise performance improvement (for review, see [17]), whereas results of the few studies assessing the effect of CAF MR remain inconclusive [12,18]. Concerning CHO MR, the main hypothesis for explaining the ergogenic effect relies upon the link between sweet tastants receptors T1Rs in the oral cavity and esophagus as well as various brain regions [19]. Supporting this idea, it has been observed that the sole presence of CHO in the mouth, prior to its ingestion, induced an increase in corticomotor pathway excitability [20]. Besides these effects in motor areas, an increase in brain activity of some areas (i.e., the dorsolateral prefrontal cortex, the orbitofrontal cortex and the striatum), known to be involved in attention and reward, has also been observed after MR [13,21]. Because of the key-role of these brain areas in executive functions, performance in cognitive tasks could also be influenced; this needs to be further investigated.

As regards to CAF MR, the rate of caffeine buccal absorption is extremely rapid and leads to a comparable amount of caffeine in the systemic circulation compared to ingestion [22]. Only very few data are available about the influence of CAF MR on cognitive performance, however it has been recently reported that CAF MR would exert a likely beneficial effect on reaction time (RT) in a task requiring executive control (Stroop task) [21]. By using electroencephalogram signal analysis, the same authors further explored this issue by assessing the effect of 20 s nasal spray with placebo, glucose and CAF on performance in the same cognitive task. While behavioral data reported no effect of nasal spray, electrophysiological data indicated that glucose and CAF nasal spray increased brain activity in some areas known to be engaged in cognitive control [23]. These brain responses could be due to the activation of bitter taste receptors T2Rs respectively in the mouth and the nasal cavity [20,21]. Further studies are required to understand the ergogenic effect of MR on exercise performance and furthermore on cognitive performance. 

The influence of nutritional supplements MR during exercise has also been investigated by measuring the rate of perceived exertion (RPE). The RPE could be defined as “the feeling of how hard, heavy and strenuous a physical task is” [24]. Results are mixed but some studies reported that CAF and/or CHO MR lead to a decrease in RPE for the same power output [25,26,27] which suggests that CHO and CAF MR may induce a decrease in subjective perception of effort, allowing participants to produce more power with the same degree of discomfort. Therefore, for all of these reasons, the ergogenic effect of nutritional substances is a topic of interest for the optimization of sport performance.

Currently, there is a growing interest for guarana (GUA), which is often attributed to CAF content depending on how the extract is prepared [28]. In addition to CAF, guarana seeds are known to harbor a number of other possible stimulants such as flavonoids [29] or other potentially psychoactive components, including saponins and tannins [30,31], which could enhance cognitive function [32,33,34]. In several studies as well as in sports nutrition strategy, GUA is rarely used alone but mostly associated to multivitamins mineral complex [32,34,35] or to ginseng [33]. We assume that, in addition to the presence of CAF, the other components of guarana seeds could potentially enhance performance via the activation of bitter taste receptors T2Rs [36] or different pathways. Some recent studies have reported that GUA ingestion could also influence cognitive abilities. More specifically, it has been reported that ingestion of GUA, could, at rest, induce an improvement in decision-making and alertness [32], improve memory performance [33] and reduce RPE after 30 min of submaximal exercise [34]. 

To the best of our knowledge, no studies have investigated the effect of MR on cognitive performance during exercise. In the field of physical activity, athletes are faced with strong physiological and cognitive demands to attain goals. Thus, the use of nutritional supplementation may ameliorate or aid to maintain a high performance and avoid adverse consequences on the outcome of sporting events. Consequently, this study aims to investigate the influence of serial administration of CHO, CAF and GUA MR on cognitive processes during an acute bout of 40-min of exercise. This exercise duration/intensity was chosen to be both appropriate to participants regularly engaged in training and enough intense to theoretically induced performance impairment (for a review see Reference [37]). Potential effects of nutritional supplements are thus expected either in terms of maintaining performance (if any impairment occurred) or ergogenic effect.

In the present study, we investigated executive functions, which in a broad sense refer to a number of processes that are necessary to remain adapted to an ever-changing environment. More specifically, we focused on cognitive control and time estimation, continuously involved in any goal-oriented behavior and we chose two well-established cognitive tasks, respectively a Simon task and a duration-production task. The Simon task [38] provides information about the ability to inhibit prepotent responses and the temporal task allows you to dissociate the effect on arousal and attention levels [39], two processes that usually remain amalgamated in most studies. The main purpose of the study was to assess whether these cognitive processes would be impacted during exercise by three different nutritional supplements commonly used by athletes and delivered by MR: CHO, CAF and guarana complex (GUAc: GUA + ginseng + vitamins C). Since results of recent studies have suggested that MR could increase brain activity in several areas that have a key-role in executive functions, we assume that both cognitive processes could benefit from supplementation during exercise.

## 2. Materials and Methods 

### 2.1. Participants

Twenty-four physically active participants (16 males and 6 females), recruited from a regional training sport center participated in this experiment without being paid (Table 1). All participants were regularly engaged in training (between 3 and 8 h per week) but were not professional cyclists. Participants were not used to drinking nutritional supplementations during training or competition and consumed less than 200 mg of caffeine per day. Before inclusion, the experimental procedure was explained to the participants and they signed an informed consent form approved by the University Ethics Committee (Ile de France, VII, Saint Germain en Laye, France). 

### 2.2. Procedure

This study used a cross over design and required the participants to visit the laboratory six times (one preliminary session, one training session and four experimental sessions) at least 72 h apart and at the same time of day. 

#### 2.2.1. Preliminary Session

One week before the start of the first experimental session, preliminary testing was performed to collect anthropometric and physiological characteristics. After a standardized warm up, the peak power output was assessed on the cycle ergometer (Cyclus 2^®^, RBM, Leipzig, Germany) using a progressive test starting at 80 Watt (W) for females and 100 W for males with an increase of 15 W/min until exhaustion. The last completed step was regarded as the peak power output (W). During the test, heart rate (HR) was continuously recorded using a monitor (RS800, Polar^®^, Helsinki, Finlande) which allows for HRmax assessment (Table 1).

#### 2.2.2. Training Session

Between 48 and 72 h prior to the first experimental session, participants underwent a training session with the duration-production task and the Simon task. Details are provided in the cognitive tasks section.

#### 2.2.3. Experimental Design

Each participant performed four experimental sessions, two per week, separated by at least 72 h and conducted at the same time of the day. Participants were instructed to keep a food diary during the two days prior to the first session and to replicate this diet before each session. They were also required to refrain from alcohol, caffeine, pain medication and nutritional supplements during the 48 h prior to each session. The last meal was taken 3 h and half prior to the start of each experimental session suggesting that participants had 3 h of fast between each session. No food or drinks intake (except water) was allowed between the last meal and the experimental session. The participants were instructed to control their time and hours of sleep the two nights preceding each experimental session. Participants were required to maintain the same training program every week during the experimental protocol. 

**Figure 1:**
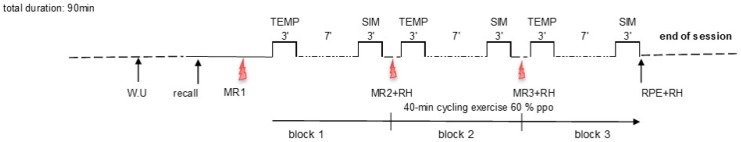
General procedure of each experimental session. (WU = Warm Up; Recall = Cognitive tasks recall; TEMP = Duration Production task; SIM = Simon task; MR = mouth rinsing; RH = Rehydration; ppo = peak power output; RPE = Rating of Perceived Exertion).

Each experimental session (see Figure 1), corresponding to a different tested nutritional mouth rinse, started with a specific cycling warm-up consisting of 2 × (2 min 100 W (for males)/80 W (for females) + 2 min 150 W (for males)/130 W (for females) and 3 × (30 s 200 W (for males)/170 W (for females) + 30 s 50 W) and lasting approximately 10 min [40]. Immediately after the warm-up, keeping the seated position on the cycle ergometer, the participants performed a short recall of the two cognitive tasks. The recall was identical to the training session except that the participants could only perform one block of training while cycling. Once the recall was made, the first mouth rinse (MR1) was administered during 20 s. Then, the cycling exercise started with a resistance fixed at 60% of the peak power output which was previously recorded for each participant. After one minute of cycling, each participant performed three identical experimental blocks separated by 20 s of MR during which they continued to cycle. Following MR2 and MR3 participants were allowed to rehydrate with as much tap water as they required. During each block, participants performed the duration-production task lasting about three minutes and seven minutes later the Simon task also lasting about three minutes. Finally, at the end of the 40-min exercise, the participants had to fill a visual analogic scale to assess RPE. Pedaling rate was freely chosen and heart rate was recorded throughout the 40 min cycling exercise using a polar RS800 cardiofrequencemeter (Polar Electro, Kempele, Finland).

Each one of the three 25 mL-MR consisted of either a 7% carbohydrate complex (CHO: fructose (89%) and maltodextrin (11%), ISOXAN^®^ Sport Pro, NHS, Rungis, France), a 67 mg caffeine (CAF, PROLAB^®^ nutrition, Chatswoth, USA) added with orange sugarless syrup, a 0.4 g guarana complex (GUA: 37.5 mg of guarana + 12.5 mg ginseng + 22.5 mg vitamins C, Isoxan Actiflash^®^ Booster, Menarini, NHS, Rungis, France), or a placebo (PL: tap water added with orange sugarless syrup) depending on the experimental session. We decided to use the amount of 7% CHO commonly used in nutrition studies and 67 mg caffeine and 37.5 mg guarana for each MR to reach the dose range of 200 mg caffeine and 300 mg guarana recommended per day.

The study was single-blind and experimental sessions were counterbalanced between participants. Each block lasted about 13 min and each session lasted about 90 min while completing the cognitive tasks for about half total time.

### 2.3. Cognitive Tasks 

The cognitive tasks were performed on a cycle ergometer, equipped with two thumb response keys fixed on the top of the right and left handle grips, positioned in front of a screen located 1m away. The cognitive tasks were first performed at rest during both the training and the recall sessions and afterward during the 40-min cycling exercise at 60% of peak power output. 

#### 2.3.1. Duration-Production Task

The duration-production task consists of pressing a button for a time interval learned during a training phase. The procedure used here was inspired from duration-production tasks used in previous studies [39,41].

##### Duration-Production Training and Recall Sessions

The training session consisted of two parts. For the first ten trials, a 600 Hz tone sounded for 1100 ms. When the sound ended, a red circle appeared in the center of the screen indicating that participants could reproduce the duration of the sound by pressing the right keypress as long as the sound lasted. When the participants released the keypress, an auditory feedback was delivered. Five different feedbacks were used. If the produced interval was correct (less than 7.5% longer or shorter than the target), the feedback “correct” was delivered. If the produced duration was too long or too short (7.5–22.5% longer or shorter than the target), either the word “too long” or “too short” were delivered. If the duration was excessively long or short (more than 22.5% longer or shorter), the words “much too long” or “much too short” were delivered. After the first ten trials, participants performed a second block in which no additional model of target duration was delivered. During the remaining trials, once the red circle appeared on the screen, participants pressed the key for 1100 ms. As in the ten first trials, an auditory feedback was delivered after each response. The participants continued until they produced 12 correct durations through 15 successive trials. A maximum of 50 trials was presented. If participants did not reach the criterion before the end of the block, they began a complete training session again (10 trials with the sound as the target duration followed by the remaining trials without the sound). 

The recall session was identical to the training session except that the participants could only perform one block of training while cycling. A recall session was performed prior to each experimental session.

##### Duration-Production Experimental Sessions

During the experimental sessions, participants were required to press the right keypress for 1100 ms. Trial onset was initiated by participants once the red circle appeared on the screen. Participants had to maintain the keypress as long as necessary to time the required duration (1100 ms). No feedback on performance was given. One and a half seconds after the release of the key, the red circle appeared again, indicating that the next trial could be initiated.

The duration-production task was performed after 60 s, 14 min and 27 min of cycling exercise and lasted approximately 3 min for each block.

#### 2.3.2. Simon Task

##### Simon Training and Recall Sessions 

In the training phase of the Simon task, all participants performed a minimum of 3 blocks of 64 trials. Two additional blocks were performed, if necessary, until the following learning criteria were achieved in one block: (a) intra-block RT variability below 5%; (b) inter-blocks RT variability with the previous block below 5%; (c) mean RT less than 400 ms; and (d) error rate between 3% and 10%. 

A short recall including 12 trials was performed at the beginning of each experimental session while cycling. No learning criterion was used.

##### Simon Task Experimental Sessions

During the whole trial, the participants had to fixate on a white point located in the center of the screen and they were required to respond, as quickly and accurately as possible, by pressing the appropriate response key (with the right or left thumb finger) according to the shape (square or circle) of a geometric symbol delivered either to the left or to the right of the fixation point. The distance from the center of the white fixation point to the center of the geometric symbol located to either the right or left was 7.5 cm. Participants had to respond according to the shape of the symbol while ignoring its spatial location. The mapping of geometric symbol shape to response key (for example, right response for a square and left response for a circle) was counterbalanced across participants. The task included two equiprobable trial types randomized: the congruent trials (CO) (response side ipsilateral to stimulus side), and the incongruent trials (IN) (response side contralateral to the stimulus side). As soon as a response key was pressed or when a delay of 1.5 s after the stimulus onset had elapsed without a response, the stimulus was removed from the screen and the next trial began. The Simon task, consisting of 64 trials, was carried out at 10 min 30 s, 23 min 30 s and 36 min 30 s during the cycling exercise. 

### 2.4. Rating of Perceived Exertion (RPE)

A Visual Analogic Scale (VAS) has been chosen since it showed a good reproducibility and sensitivity to assess the subjective effort during exercise [42]. The scale was used under the form of a vertical line, 20 cm of length, anchored by word descriptors on the top (maximal exertion) and on the bottom (no exertion at all) to gauge how hard was the exercise. At the end of the experimental session, participants marked a point on the line that they felt represented the intensity of exercise at the end of the cycling session. The VAS score was determined by the distance in centimeters from the bottom line to the point marked by the participants.

### 2.5. Statistical Analysis

Results were analyzed with quantitative (General Linear Models, GLM) or qualitative (probabilistic magnitude-based inferences) methods. Firstly, repeated measures within participants GLM were performed on each dependent variable for exercise effect (mean RT, error rate, mean produced duration, temporal variance) and post-hoc Newman-Keuls analyses were conducted for all significant effects. Significance was set at *p* < 0.05 for all analyses. Effect sizes for GLM were calculated using partial eta square (*ɳ_p_*^2^). Secondly, to specifically test effects of nutritional supplements and RPE, we also reported probabilistic magnitude-based inferences about all the variables using methods described by Hopkins et al. [43] which has been applied in several recent nutritional studies [21,44,45]. Data were log-transformed prior to analyses to reduce bias arising from non-uniformity of error. To compare within-trial changes between trials, we used a modified statistical spreadsheet that calculates the between-trials differences or effect sizes (ES), 90% confidence interval (CI) using the pooled standard deviation. If the chance of benefit or harm were both >5% the true effect was reported as unclear. Otherwise, chances of benefit or harm were assessed as follows: <1%, almost certainly not; 1–5%, very unlikely; 5–25% unlikely; 25–75% possible; 75–95% likely; 95–99% very likely; >99% most likely [46]. 

## 3. Results

The two cognitive tasks were assessed during a 40-min cycling exercise at 60% of peak power output. Heart rate during exercise increased to 78% ± 1.2% HRmax in block 1, 82.3% ± 1.3% HRmax in block 2 and 83.8% ± 1.2% HRmax in block 3.

### 3.1. Cognitive Performance

An overview of cognitive results is shown in Table 2.

#### 3.1.1. Duration-Production Task

Mean produced duration, measuring the accuracy and variance of produced durations, were calculated for each participant in each of the four conditions. Mean produced duration reflects overall lengthening or shortening of produced time. To aid in the interpretation of results, it should be noted that a shortening of produced durations (also called underproduction) means that the criterion value corresponding to the duration learned during the training session was reached earlier. Conversely, a lengthening of produced durations (also called overproduction) means that the criterion value was reached later [41]. The variance of produced durations provides information about individual participant’s variability across trials. The mean and the variance of the produced duration were submitted to separate GLM with block (block 1, block 2 and block 3) as within-subject factors and to magnitude-based inferences for the effects of nutritional supplements.

##### Mean in Temporal Production

**Figure 2:**
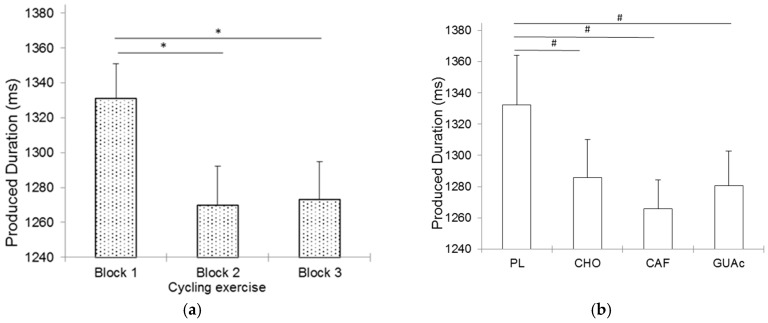
Mean produced durations as a function of exercise (**a**) and supplementation (**b**) Errors bars represent standard errors of the mean produced duration. * *p* < 0.05; ^#^ 75–95% likely different.

A significant effect of block was observed on mean produced duration (*F*(2, 44) = 7.6, *p* = 0.001, *ɳ_p_*^2^ = 0.26). Participants produced shorter durations in blocks 2 and 3 than in block 1 (respectively *p* = 0.003 and *p* = 0.003) (Figure 2a). Thus, although participants globally overproduced the target 1100 ms duration, they underproduced duration when completing the cycling exercise. 

Nonetheless, magnitude based inferences showed that CHO, CAF and GUAc MR compared to PL lead to a likely decrease on mean produced durations (respectively 80%, 86% and 81%), which would mean that participants globally underproduced duration after MR with CHO, CAF and GUAc when compared with PL (Figure 2b).

##### Variance in Temporal Production

A significant effect of block was observed on the variability (*F*(2, 44) = 4.68, *p* = 0.01, *ɳ_p_*^2^ = 0.18). The participants were significantly less variable in block 3 in comparison to blocks 1 and 2 (respectively *p* = 0.01 and *p* = 0.05). 

Magnitude based inferences showed that CHO, CAF and GUAc MR compared to PL, lead to a likely decrease of variability (respectively 93%, 89% and 92%). Participants were less variable in their temporal production after nutritional supplementation. Furthermore, they were even less variable with CHO when compared to GUAc (likely effect). 

#### 3.1.2. Simon Task

The mean RT and the error rate were submitted to separate GLM block (block 1, block 2 and block 3) and congruency (CO and IN) as within-subject factors. Nutritional effect was evaluated with magnitude-based inferences.

##### Mean RT 

Analyses showed a significant effect of exercise on mean RT (*F*(2, 44) = 10.1, *p* = 0.0003, *ɳ_p_*^2^ = 0.31). RTs in Block 3 are significantly faster than RTs in blocks 1 and 2 respectively (*p* = 0.001 and *p* = 0.002). The classic effect of congruency was found on mean RTs (*F*(1, 22) = 78.3, *p* = 0.00, *ɳ_p_*^2^ = 0.78) with slower RT in IN trials compared to RT in CO trials. 

**Figure 3:**
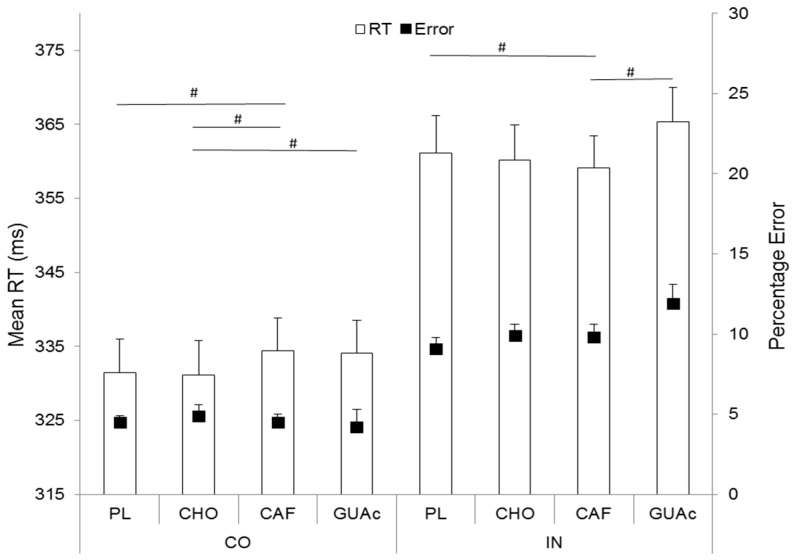
Mean reaction time (RT) and errors as a function of supplementation and congruency. Errors bars represent standard errors of mean RT. # 75–95% likely different.

**Figure 4:**
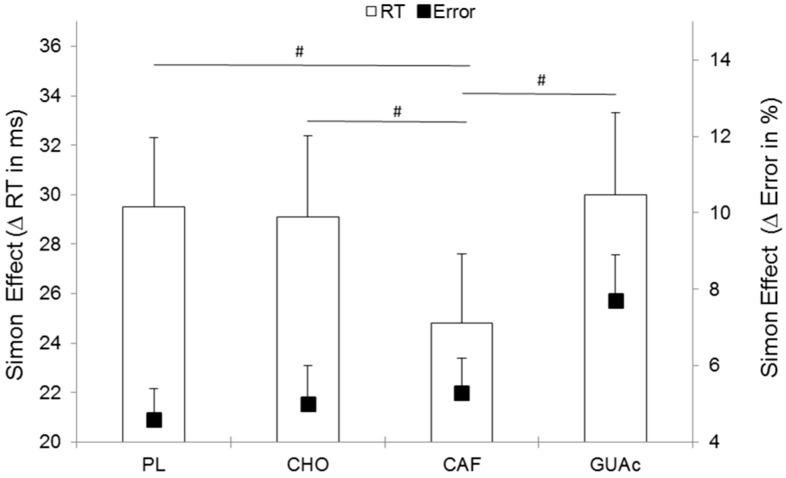
Simon Effect for reaction time (RT) and error as a function of supplementation. Errors bars represent standard errors of the mean Δ. # 75–95% likely different.

For CO trials, magnitude based inference showed that CAF leads to a likely increase of mean RT compared with PL (80%) and CHO (82%), and that CHO leads to a likely decrease of RT compared with GUAc (89%). On IN trials, CAF leads to a likely decrease of mean RT compared to PL (90%) and GUAc (94%) (Figure 3). More interestingly, magnitude based inference on the Simon effect (i.e., difference between mean IN RT and mean CO RT), which is a direct measure of cognitive control, showed a likely decrease with CAF MR (24 ms) when compared with PL (30 ms, 93%), CHO (29 ms, 85%) and GUAc (79%) (Figure 4). 

##### Error Rate

Analyses showed a significant effect of congruency (*F*(1, 22) = 33.44 *p* = 0.00, *ɳ_p_*^2^ = 0.60), illustrating the prevalence of more errors in IN trials than in CO trials. The effect of block tended to be significant (*F*(2, 44) = 3.8, *p* = 0.07, *ɳ_p_*^2^ = 0.11). Errors in block 3 tended to be significantly lower than in block 1 (*p* = 0.06). No significant difference between block 2 and respectively blocks 1 (*p* = 0.11) and 3 (*p* = 0.47) was found. 

Magnitude based inference did not show any effect of nutritional supplementations on error rate.

### 3.2. RPE

Magnitude based inferences showed that CHO MR lead to a likely decrease in RPE (11.6 ± 0.7) cm when compared to PL ((12.2 ± 0.8) cm; 87%), GUAc ((12.5 ± 0.8) cm; 91%) and CAF ((12.3 ± 0.7) cm; 91%).

## 4. Discussion

The aim of this study was to investigate the influence of serial CHO, GUAc and CAF MR both on cognitive performance during a 40-min cycling exercise at a submaximal intensity and on RPE at the end of the exercise. Specific cognitive processes, such as cognitive control and time perception, considered as being of great importance in goal-oriented behavior, have been investigated through a Simon task and a duration-production task. Several major findings have emerged from this study: 1/CHO, CAF and GUAc MR likely lead participants to produce shorter durations and to reduce temporal variability, 2/CAF MR likely improved cognitive control and CHO MR led to a likely decrease in RPE after exercise. Moreover, performing 40-min of submaximal exercise enhanced information processing in terms of both speed and accuracy, decreased the variance in temporal production, led participants to produce shorter durations and did not alter cognitive control.

### 4.1. Effect of Physical Exercise on Cognitive Performance

Although the effects of exercise on cognition were not the main purpose, innovative results emerged from this study. It therefore seemed interesting to discuss it in more detail in the first part of the discussion. It is now classically reported that exercise has a positive effect on cognitive tasks, however, cognitive performance has typically been assessed during short duration exercise (about 20 min) or immediately after exercise while limited research has investigated cognition during longer exercises [47]. In the present study, we observed performance improvement in cognitive tasks throughout the exercise. In the duration-production task, participants produced shorter durations after 14 min, and they were less variable after 27 min of cycling exercise when compared to the beginning of the exercise. Most of the models that have been proposed to explain performance in temporal tasks are based on scalar timing theory [48,49]. According to this theory, people are supposed to estimate time intervals using an internal clock functioning as a stopwatch, with a clock stage composed of a pacemaker-counter device. An interval is specified by the accumulation of pulses emitted at a regular rate from the pacemaker. The more pulses that are accumulated, the longer the subjective estimation of duration. Within this framework, one hypothesis to explain shorter produced durations (called underproductions) is an acceleration of the pacemaker rate [39,50,51]. When pacemaker rate increases, pulses are emitted and accumulated faster. The number of pulses corresponding to the standard duration they have learned during the training session, would then be accumulated in a shorter time interval, leading to the production of shorter durations. The present data thus suggest that submaximal exercise leads to an acceleration of the pacemaker rate. In literature investigating the psychology of time, several studies show that the acceleration of the pacemaker rate is related to an increase in cortical arousal [39,50]. Moreover, we have observed a decrease in temporal variability, which is also classically explained by an increase in alertness level [52]. This increase in cortical arousal could be induced by an enhancement of the noradrenergic and dopaminergic systems during exercise [53,54]. During exercise the sympathetic nervous system is rapidly stimulated, leading to large increases in the secretion of adrenaline and noradrenaline from the adrenal medulla and noradrenaline from sympathetic nerves. Even if catecholamines do not cross the blood brain barrier [55], evidence has shown that a peripheral circulating catecholamines increase during exercise is related, by several mechanisms, to an increase in concentrations of brain catecholamines, which activate the prefrontal cortex and other associated areas [56,57].

After 23 min of cycling, a positive effect of exercise was also observed in the Simon task in which we observed faster RTs without an increase of error rate (trend to lower error rates from the 13th min of exercise is even observed). Several meta-analyses and integrative reviews report that cognitive performance can be improved during acute exercise depending on factors like physical exercise intensity and duration, physical fitness of participants and the nature of the cognitive tasks [47,58,59]. This benefit can be explained by an enhancement of sensory sensibility [60], a better efficiency of peripheral motor processes [61,62] and/or a reduction in intracortical inhibition accompanied by an increase in corticospinal excitability [53]. The results of the present study are in line with these findings. Indeed, both the improvement of the information processing and the temporal variability and the underproduction of durations, suggest a possible increase in the brain activation level, which would result from an activation of the reticular-activating system [54]. 

However, cognitive processes seem to be differentially sensitive to the effect of exercise. To date, the accumulated evidence is equivocal and provides an unclear picture of the relationship between exercise intensity and cognitive control. Despite some evidence that suggests an impairment of cognitive control (using a Simon task or a similar conflict task) during exercise [63,64], others [65,66] fail to observe any deteriorations of selective response inhibition or rather report an improvement despite very high physiological stress [67]. In this study, the lack of exercise effect on the interference effect (as shown by the absence of significant block x congruency interaction) suggested that the ability to inhibit prepotent responses remains fully efficient during exercise. 

### 4.2. Mouth Rinse Effect on Cognitive Performance

Concerning the MR effects on cognitive performance, magnitude based inferences showed that CHO, CAF and GUAc MR likely lead to shorter produced durations and a decrease in temporal production variability. As previously noticed, temporal underproductions may be explained by an acceleration of the pacemaker rate due to an increase in arousal level [39,50]. This increase of cortical arousal is also consistent with an acceleration in RTs. RTs were likely shorter in the IN trials (with no variation in the error rate) after CAF MR compared to PL and GUAc MR. This improvement resulted in a decrease in the Simon effect (or interference effect), which is a direct measure of cognitive control and highlighted an improvement in cognitive performance. 

The ergogenic effect of CAF on physical and cognitive performance has already been reported after ingestion [3,68]. It has been proposed that it could be at least partially related to competitive inhibition of adenosine receptors [69]. The effects of CHO ingestion on cognitive performance have not been thoroughly investigated but some studies report a positive effect on cognition. For example, a positive effect of CHO ingestion has been observed on RT after exercise [70,71]. Moreover, Lieberman and colleagues [72] found an improvement in vigilance and mood during sustained physical activity with CHO ingestion. The hypothesis is that CHO intake may affect cognition by modulating the synthesis of certain neurotransmitters. The most cited are acetylcholine, which is dependent upon glucose for its synthesis, and brain serotonin, a neurotransmitter associated with sleepiness (for review, see Reference [69]). 

The effect of nutritional MR without ingestion was more specifically investigated in the present study. Several studies highlighted that CHO and CAF MR could have a positive effect on physical [12,17,18,25,73,74] and on cognitive performance [13,15,21]. The present results showed that both CHO and CAF MR lead to underproduced durations and a reduction of temporal variability both considered to be due to an increase in cortical activation level in the field of the psychology of time [39,50]. This is consistent with data from electroencephalographic activity (EEG) reported by de Pauw and collaborators [21]. Measures of cortical brain activity were regarded as an index of cortical arousal and served as a sensitive indication of the stimulating effects of CAF on brain functioning. Results report that CAF MR exert a likely beneficial effect on RT in IN trials only in a Stroop Task which is entirely consistent with our results showing that CAF MR only lead to shorter RT with no errors shift in IN trials. The authors proposed that the observed effect was due to the subsequent activation of both orbitofrontal and dorsolateral prefrontal cortices. They also observed that CHO MR induced a higher brain activity within the orbitofrontal cortex but had no effect on RT.

Concerning GUAc MR, we found some effects in the temporal task. Participants underproduced durations after GUAc MR compared with PL MR, which suggests that GUAc MR increased brain activation level as did CHO and CAF MR. To our knowledge, this is the first time that the effect of GUA MR was observed on cognitive performance. The duration-production task is known to be highly sensitive to arousal manipulation, which could explain why data exhibited no effect in the Simon task.

Finally, magnitude based inferences have also revealed that CHO MR leads to a likely decrease in RPE for the same power output compared to PL. This result is very interesting since results are mixed and most studies have failed to observe benefits from CHO MR [9,44,75,76,77] and CAF MR [18,78] on perception of effort. Only a few studies have reported that CAF and CHO MR could lead to a decrease in RPE [25,26,27] suggesting that CHO and CAF MR induce a decrease in subjective perception of effort, allowing participants to produce more power with the same degree of discomfort. This finding may be explained by the activation of reward areas reported by Silva et al. [17] via stimulation of CHO receptors in the mouth. Indeed, the activation of the insula/frontal operculum, orbitofrontal cortex and striatum, could result in a lowering perceived exertion during exercise. Chambers (2009) [13] suggested that the activation of these brain areas could modify fatigue signals sent from the muscles and result in lower subjective perception of effort for the same power output. Concerning GUAc, our results failed to highlight a positive effect on RPE as observed in a recent study using GUAc ingestion [34]. The authors observed a small but significant reduction in RPE during a 30-min treadmill running exercise after ingestion of multivitamin and mineral complex with guarana. The difference of results could be due, first, to the lower amount of GUAc used in our study compared to Veasey and colleagues’ study [34] and, second, to the different administration modes (ingestion versus MR). We can hypothesize that with MR, a larger amount of GUA would be required to impact the RPE. 

One limitation of the present study is the use of a multivitamin and mineral complex with guarana instead of GUA solely, which did not allow isolate the pure effect of GUA MR on performance. At present, mechanisms accountable of ergogenic effects of GUA ingestion on cognitive performance need to be further investigated. We should also note that some studies suggest that oral contraceptive steroids and estrogen have a significant impact on caffeine metabolism [79] and that RT lengthens in the high-progesterone, luteal phase of the menstrual cycle [80]. The fact that the influence of different phases of menstrual cycle of the female participants has not been controlled in the present study, due to methodological difficulties, is also a limitation. Further controlled studies will be necessary to examine potential interactions with mouth rinsing.

Magnitude based inferences showed that CHO, CAF and GUAc MR enhance performance. It is still important to note that nutritional supplements benefited only half of the participants. However, when a beneficial effect of MR was observed it was up to two to three times greater than the deterioration (or lack of effect) observed on the other half of the subjects. These findings suggest that, as for ingestion of nutritional supplementations, the effects of MR on performance are highly variable between individuals. Before using nutritional strategies with athletes during sporting events, it seems essential to determine beforehand whether the athlete is a good or bad responder. 

## 5. Conclusions

The present study was the first to investigate the effects of different nutritional supplements MR on cognitive performance during exercise. Our data have suggested that the serial administration of CHO, CAF and GUAc MR improves cognitive performance and decreases subjective perception of effort. Moreover, they confirm that submaximal exercise increased arousal levels leading to an improvement in cognitive performance [53,54]. The present study provided important new insights regarding the specific changes in cognitive processes induced by nutritional supplements and opened up new perspectives regarding the use of MR to optimize cognitive performance during exercise. Obviously, some further studies are needed to confirm these results and to more investigate specific cognitive processes in more depth. Moreover, while the present study involved a 40-min cycling exercise at a submaximal intensity, which is longer than, most of the previous studies, future studies involving more exhausting exercises, in terms of duration and/or intensity, are needed to further elucidate the relationships between nutrition supplements and fatigue and to uncover whether interactions between these two factors could emerge. 

## Figures and Tables

**Table 1:** Anthropometrical and physiological characteristics of participants.

Mean (Standard Deviation)			
Variables	All	Female	Male
Sample size	24	8	16
Age (years)	26 (8)	30 (10)	24 (6)
Height (cm)	174 (10)	164 (7)	179 (7)
Body mass (kg)	72 (14)	57 (7)	79 (11)
Body mass index (kg·m^−2^)	23 (3)	21 (2)	25 (3)
Peak power output (W)	250 (58)	198 (31)	276 (50)
Cycling intensity experimental sessions (W)	150 (35)	119 (18)	166 (30)
Heart rate max (bpm·min^−1^)	189 (8)	185 (10)	190 (7)

**Table 2:** Cognitive performance as a function of nutritional supplementation and exercise.

			Duration Production Task Mean (Standard Errors)		Simon Task Mean (Standard Errors)							
			Produced Duration (ms)	Variance (ms)	Reaction Time (ms)				Errors (%)			
					CO	IN	Total	Simon Effect §	CO	IN	Total	Simon Effect §
Nutritional supplementation	PL	Block 1	1370.7 (50.9)	197.9 (17.5)	336.0 (8.7)	365.5 (9.7)	350.7 (6.8)	29.4 (5.7)	4.7 (0.8)	9.8 (1.2)	7.2 (0.8)	5.1 (1.2)
		Block 2	1313.3 (56.8)	201.4 (24.0)	334.3 (7.3)	359.8 (9.0)	347.1 (6.0)	25.5 (5.1)	4.3 (0.9)	8.9 (1.3)	6.6 (0.9)	4.6 (1.3)
		Block 3	1312.7 (58.6)	185.6 (15.9)	323.9 (7.7)	357.6 (7.9)	340.8 (6.0)	33.7 (3.7)	4.6 (0.6)	8.7 (1.2)	6.6 (0.7)	4.2 (1.5)
		Total	1332.2 (31.8)	195.0 (11.1)	331.4 (4.6) ●	361.1 (5.1) ●	346.2 (3.6)	29.5 (2.8) ●	4.5 (0.4)	9.1 (0.7)	6.8 (0.5)	4.6 (0.8)
	CHO	Block 1	1348.9 (44.2)	186.8 (14.5)	334.7 (8.1)	362.2 (8.0)	348.4 (6)	27.5 (6.3)	7.0 (1.5)	10.4 (1.7)	8.7 (1.2)	3.4 (2.6)
		Block 2	1259.2 (41.0)	171.6 14.6)	334.4 (8.4)	361.8 (8.5)	348.1 (6.1)	27.5 (5.5)	3.3 (0.8)	9.5 (0.9)	6.4 (0.8)	6.3 (1.0)
		Block 3	1249.6 (39.4)	161.5 (11.8)	324.2 (8.0)	356.5 (8.1)	354.6 (6.1)	32.3 (5.5)	4.4 (0.9)	9.8 (1.1)	7.1 (0.8)	5.3 (1.2)
		Total	1285.9 (24.3) **◘**	173.3 (7.9) ◘ ▲	331.1 (4.7) ●	360.2 (4.7)	345.6 (3.5)	29.1 (3.3) ●	4.9 (0.7)	9.9 (0.7)	7.4 (0.5)	5.0 (1.0)
	CAF	Block 1	1292.6 (24.5)	187.0 (11.8)	338.0 (8.5)	364.4 (8.9)	351.2 (6.3)	26.4 (5.12)	5.1 (0.9)	10.3 (1.3)	7.7 (0.9)	5.2 (1.6)
		Block 2	1255.9 (39.1)	187.3 (14.3)	336.8 (8.0)	360.7 (6.7)	348.8 (5.5)	23.9 (4.6)	3.9 (0.9)	10.5 (1.4)	7.2 (0.9)	6.6 (1.6)
		Block 3	1249.0 (31.5)	167.7 (11.5)	328.4 (7.4)	352.4 (7.4)	340.4 (5.5)	24 (4.7)	4.6 (0.8)	8.5 (1.4)	6.5 (0.9)	3.9 (1.7)
		Total	1265.8 (18.5) **◘**	180.7 (7.3) ◘	334.4 (4.6)	359.1 (4.4)	346.8 (3.3)	24.8 (2.8)	4.5 (0.5)	9.8 (0.8)	7.1 (0.5)	5.3 (0.9)
	GUA	Block 1	1311.2 (35.4)	193.7 (15.8)	338.7 (7.9)	370.4 (8.7)	354.6 (6.3)	31.7 (6.4)	5.8 (1.1)	10.5 (1.5)	8.2 (1.0)	4.6 (1.8)
		Block 2	1249.9 (40.4)	176.5 (13.1)	336.8 (7)	365.8 (7.9)	351.3 (5.7)	29 (5.6)	3.8 (0.9)	13.3 (2.3)	8.6 (1.4)	9.5 (2.6)
		Block 3	1281.1 (40.2)	170.5 (13.2)	326.9 (7.9)	360.1 (7.6)	343.5 (6.0)	33.2 (5.4)	3.0 (0.7)	12.0 (1.6)	7.5 (1.1)	9.0 (2.8)
		Total	1280.7 (22.2) ◘	180.2 (8.1) ◘	334.1 (4.4) ■	365.4 (4.6) ●	349.8 (3.4)	30.0 (3.3) ●	4.2 (0.6)	11.9 (1.1)	8.1 (0.7)	7.7 (1.2)
Exercise		Block 1	1331.0 (20.0) **$**	191.3 (7.4) **$**	336.9 (4.1)	365.5 (4.4)	351.2 (3.2) **$**	28.4 (2.9)	5.7 (0.6)	10.2 (0.7)	8.0 (0.5)	8.0 (0.5)
		Block 2	1269.8 (22.3) *	184.3 (8.5) **$**	335.6 (3.8)	362.0 (4.0)	348.8 (2.9) **$**	26.2 (2.6)	3.8 (0.4)	10.5 (0.8)	7.1 (0.5)	7.1 (0.5)
		Block 3	1273.0 (21.7) *	171.3 (6.6) *	325.9 (3.8)	356.6 (3.8)	340.9 (2.9)	30.5 (2.4)	4.1 (0.4)	9.7 (0.7)	6.8 (0.4) ~	6.8 (0.4)
		Total	1292.2 (12.4)	182.9 (4.4)	332.9 (2.4)	361.4 (2.3)	347.2 (2.0)	29.95 (1.8)	4.5 (0.3)	10.2 (0.4)	7.4 (0.3)	5.6 (0.5)

§Incongruent (IN) minus congruent (CO) trials. General Linear Models (GLM): * a significant difference was found with block 1; ^$^ a significant difference was found with block 3; ^~^ a trend effect was found with block 1 (*p* < 0.05). Magnitude based inference: ^◘^ a difference was found with placebo mouth rinsing, PL MR; ^▲^ a difference was found with guarana mouth rinsing, GUA MR; ^●^ a difference was found with caffeine mouth rinsing, CAF MR; ^■^ a difference was found with carbohydrate mouth rinsing, CHO MR (75–95% likely different).

## Supplementary Materials

The following are available online at www.mdpi.com/2072-6643/9/6/589/s1.

## References

[1] Close G.L., Hamilton D.L., Philp A., Burke L.M., Morton J.P. (2016). New strategies in sport nutrition to increase exercise performance. Free Radic. Biol. Med..

[2] Vandenbogaerde T.J., Hopkins W.G. (2011). Effects of Acute Carbohydrate Supplementation on Endurance Performance. Sports Med..

[3] Glade M.J. (2010). Caffeine-Not just a stimulant. Nutrition.

[4] Glaister M., Howatson G., Abraham C.S., Lockey R.A., Goodwin J.E., Foley P., McInnes G. (2008). Caffeine supplementation and multiple sprint running performance. Med. Sci. Sports Exerc..

[5] Hogervorst E., Bandelow S., Schmitt J., Jentjens R., Oliveira M., Allgrove J., Carter T., Gleeson M. (2008). Caffeine improves physical and cognitive performance during exhaustive exercise. Med. Sci. Sports Exerc..

[6] McNaughton L.R., Lovell R.J., Siegler J., Midgley A.W., Moore L., Bentley D.J. (2008). The effects of caffeine ingestion on time trial cycling performance. Int. J. Sports Physiol. Perform..

[7] Graham T.E. (2001). Caffeine and exercise: Metabolism, endurance and performance. Sports Med..

[8] Cermak N.M., van Loon L.J.C. (2013). The Use of Carbohydrates during Exercise as an Ergogenic Aid. Sports Med..

[9] Carter J.M., Jeukendrup A.E., Jones D.A. (2004). The effect of carbohydrate mouth rinse on 1-h cycle time trial performance. Med. Sci. Sports Exerc..

[10] Jeukendrup A.E., Brouns F., Wagenmakers A.J.M., Saris W.H.M. (1997). Carbohydrate-Electrolyte Feedings Improve 1 h Time Trial Cycling Performance. Int. J. Sports Med..

[11] Jeukendrup A.E. (2013). Oral Carbohydrate rinse: Placebo or beneficial?. Curr. Sports Med. Rep..

[12] Beaven C.M., Maulder P., Pooley A., Kilduff L., Cook C. (2013). Effects of caffeine and carbohydrate mouth rinses on repeated sprint performance. Appl. Physiol. Nutr. Metab..

[13] Chambers E.S., Bridge M.W., Jones D.A. (2009). Carbohydrate sensing in the human mouth: Effects on exercise performance and brain activity. J. Physiol..

[14] James R.M., Ritchie S., Rollo I., James L.J. (2016). No Dose Response Effect of Carbohydrate Mouth Rinse on Cycling Time Trial Performance. Int. J. Sport Nutr. Exerc. Metab..

[15] Philips S.M., Findlay S., Kavaliauskas M., Grant M.C. (2014). The Influence of Serial Carbohydrate Mouth Rinsing on Power Output during a Cycle Sprint. J. Sports Sci. Med..

[16] Pottier A., Bouckaert J., Gilis W., Roels T., Derave W. (2010). Mouth rinse but not ingestion of a carbohydrate solution improves 1-h cycle time trial performance. Scand. J. Med. Sci. Sports.

[17] E Silva T.A., Di Cavalcani Aves de Souza M.E., de Amorin J.F., Stathis C.G., Leandro C.G., Lima-Silva A.E. (2014). Can Carbohydrate Mouth Rinse Improve Performance during Exercise? A systematic Review. Nutrients.

[18] Doering T.M., Fell J.W., Leveritt M.D., Desbrow B., Shing C.M. (2014). The effect of a caffeinated mouth-rinse on endurance cycling time-trial performance. Int. J. Sport Nutr. Exerc. Metab..

[19] Turner C.E., Byblow W.D., Stinear C.M., Gant N. (2014). Carbohydrate in the mouth enhances activation of brain circuitry involved in motorperformance and sensory perception. Appetite.

[20] Gam S., Guelfi K.J., Fournier P.A. (2016). New Insights into enhancing maximal exercise performance through the use of bitter tastant. Sports Med..

[21] De Pauw K., Roelands B., Knaepen K., Polfiet M., Stiens J., Meeusen R. (2015). Effects of caffeine and maltodextrin mouth rinsing on P300, brain imaging and cognitive performance. J. Appl. Physiol..

[22] Kaminori G.H., Karyekar C.S., Otterstetter R., Cox D.S., Balkin T.J., Belenky G.L., Eddington N.D. (2002). The rate of absorption and relative bioavailability of caffeine administered in chewing gum versus capsules to normal healthy volunteers. Int. J. Pharm..

[23] De Pauw K., Roelands B., van Cutsem J., Marusic U., Torbeyns T., Meeusen R. (2016). Electro-physiological changes in the brain induced by caffeine or glucose nasal spray. Psychopharmacology.

[24] Pageaux B. (2016). Perception of effort in Exercise Science: Definition, measurement and perspectives. Eur. J. Sport Sci..

[25] Bastos-Silva V.J., Melo A.A., Lima-Silva A.E., Moura F.A., Bertuzzi R., de Araujo G.G. (2016). Carbohydrate Mouth Rinse Maintains Muscle Electromyographic Activity and Increases Time to Exhaustion during Moderate but not High-Intensity Cycling Exercise. Nutrients.

[26] Fares E.J.M., Kayser B. (2011). Carbohydrate mouth rinse effects on exercise capacity in pre-post prandial states. J. Nutr. Metab..

[27] Sinclair J., Bottoms L., Flynn C., Bradley E., Alexander G., McCullagh S., Finn T., Hurst H.T. (2014). The effect of different durations of carbohydrate mouth rinse on cycling performance. Eur. J. Sport Sci..

[28] Schimpl F., Silva J., Goncalves J., Mazzafera P. (2013). Guarana: Revisiting a highly caffeinated plant from the Amazon. J. Ethnopharmacol..

[29] Scholey A., Haskell C. (2008). Neurocognitive effects of guaraná plant extract. Drugs Futur..

[30] Espinola E.B., Dias R.F., Mattei R., Carlini E.A. (1997). Pharmacological activity of Guarana (*Paullinia cupana* Mart.) in laboratory animals. J. Ethnopharmacol..

[31] Mattei R., Dias R.F., Espínola E.B., Carlini E.A., Barros S.B. (1998). Guarana (*Paullinia cupana*): Toxic behavioral effects in laboratory animals and antioxidants activity in vitro. J. Ethnopharmacol..

[32] Pomportes L., Davranche K., Brisswalter I., Hays A., Brisswalter J. (2014). Heart Rate Variability and Cognitive Function Following a multi-vitamin and Mineral Supplementation with added Guarana (*Paullinia cupana*). Nutrients.

[33] Kennedy D.O., Haskell C.F., Wesnes K.A., Scholey A.B. (2004). Improved cognitive performance in human volunteers following administration of guarana (*Paullinia cupana*) extract: Comparison and interaction with Panax ginseng. Pharmacol. Biochem. Behav..

[34] Veasey R.C., Haskell-Ramsay C.F., Kennedy D.O., Wishart K., Maggini S., Fuchs C.J., Stevenson E.J. (2015). The Effects of Supplementation with a Vitamin and Mineral Complex with Guaraná Prior to Fasted Exercise on Affect, Exertion, Cognitive Performance, and Substrate Metabolism: A Randomized Controlled Trial. Nutrients.

[35] Kennedy D.O., Haskell C.F., Robertson B., Reay J., Brewster-Maund C., Luedemann J., Maggini S., Ruf M., Zangara A., Scholey A.B. (2008). Improved cognitive performance and mental fatigue following a multi-vitamin and mineral supplement with added guaraná (*Paullinia cupana*). Appetite.

[36] Soares S., Kohl S., Thalmann S., Mateus N., Meyerhof W., de Freitas V. (2013). Different phenolic compounds activate distinct human bitter taste receptors. J. Agric. Food Chem..

[37] Millet G.Y., Lepers R. (2004). Alterations of neuromuscular function after prolonged running, cycling and skiing exercises. Sports Med..

[38] Simon J.R., Rudell A.P. (1967). Auditory S-R compatibility: The effect of an irrelevant cue on information processing. J. Appl. Psychol..

[39] Burle B., Casini L. (2001). Dissociation between Activation and Attention Effects in Time Estimation: Implications for Internal Clock Models. J. Exp. Psychol. Hum. Percept. Perform..

[40] McGowan C.J., Pyne D.B., Thompson K.G., Rattray B. (2015). Warm-Up Strategies for Sport and Exercise: Mechanisms and Applications. Sports Med..

[41] Casini L., Ramdani-Beauvir C., Burle B., Vidal F. (2013). How does one night of sleep deprivation affect the internal clock?. Neuropsychologia.

[42] Ueda T., Nabetani T., Teramoto K. (2006). Differential perceived exertion measured using a new visual analogue scale during pedaling and running. J. Physiol. Anthropol..

[43] Hopkins W.G., Marshall S.W., Batterham A.M., Hanin J. (2008). Progressive Statistics for Studies in Sports Medicine and Exercise Science. Med. Sci. Sports Exerc..

[44] Luden N.D., Saunders M.J., D’Lugos A.C., Pataky M.W., Baur D.A., Vining C.B., Schroer A.B. (2016). Carbohydrate Mouth Rinsing Enhances High Intensity Time Trial Performance Following Prolonged Cycling. Nutrients.

[45] Pataky M.W., Womack C.J., Saunders M.J., Goffe J.L., D’Lugos A.C., El-Sohemy A., Luden N.D. (2016). Caffeine and 3-km cycling performance: Effects of mouth rinsing, genotype, and time of the day. Med. Sci. Sports Exerc..

[46] Le Meur Y., Pichon A., Schaal K., Schmidtt L., Louis J., Gueneron J., Vidal P.P., Hausswirth C. (2013). Evidence of Parasympathetic Hyperactivity in Functionally Overreached Athletes. Med. Sci. Sports Exerc..

[47] Lambourne K., Tomporowski P. (2010). The effect of exercise-induced arousal on cognitive task performance: A meta-regression analysis. Brain Res..

[48] Gibbon J. (1977). Scalar expectancy theory and Weber’s law in animal timing. Psychol. Rev..

[49] Gibbon J., Church R.M., Meck W.H. (1984). Scalar Timing in Memory. Ann. N. Y. Acad. Sci..

[50] Penton-Voak I.S., Edwards H., Percival A., Wearden J.H. (1996). Speeding up an internal clock in humans? Effects of click trains on subjective duration. J. Exp. Psychol. Anim. Behav. Process..

[51] Wearden J.H., Edwards H., Fakhri M., Percival A. (2008). Slowing down an internal clock: Implications for accounts of performance on four timing tasks. Q. J. Exp. Psychol..

[52] Droit-Volet S. (2003). Alerting attention and time perception in children. J. Exp. Child Psychol..

[53] Davranche K., Temesi J., Verges S., Hasbroucq T. (2015). Transcranial magnetic stimulation probes the excitability of the primary motor cortex: A framework to account for the facilitating effects of acute whole-body exercise on motor processes. J. Sport Health Sci..

[54] Dietrich A., Audiffren M. (2011). The reticular-activating hypofrontality (RAH) model of acute exercise. Neurosci. Biobehav. Rev..

[55] Kostrzewa R.M. (2007). The blood-brain barrier for catecholamines—Revisited. Neurotox. Res..

[56] McMorris T., Hale B.J., Corbett J., Robertson K., Hodgson C.I. (2015). Does acute exercise affect the performance of whole-body, psychomotor skills in an inverted-U fashion? A meta-analytic investigation. Physiol. Behav..

[57] Tank A.W., Lee W.D. (2015). Peripheral and central effects of circulating catecholamines. Compr. Physiol..

[58] Brisswalter J., Collardeau M., Arcelin R. (2002). Effects of acute physical exercise on cognitive performance. Sports Med..

[59] Tomporowski P.D. (2003). Effects of acute bouts of exercise on cognition. Acta Psychol..

[60] Davranche K., Pichon A. (2005). Critical flicker frequency thresholds increment after exhanding exercise. J. Sport Exerc. Psychol..

[61] Davranche K., Burle B., Audiffren M., Hasbroucq T. (2005). Information processing during physical exercise: A chronometric and electromyographic study. Exp. Brain Res..

[62] Davranche K., Burle B., Audiffren M., Hasbroucq T. (2006). Physical exercise facilitates motor processes in simple reaction time performance: An electromyographic analysis. Neurosci. Lett..

[63] Davranche K., Paleresompoulle D., Pernaud R., Labarelle J., Hasbroucq T. (2009). Decision making in elite white-water athletes paddling on a kayak ergometer. J. Sport Exerc. Psychol..

[64] McMorris T., Davranche K., Jones G., Hall B., Corbett J., Minter C. (2009). Acute incremental exercise, performance of a central executive task, and sympathoadrenal system and hypothalamic-pituitary-adrenal axis activity. Int. J. Psychophysiol..

[65] Davranche K., Brisswalter J., Radel R. (2015). Where are the limits of the effects of exercise intensity on cognitive control?. J. Sport Health Sci..

[66] Davranche K., Hall B., McMorris T. (2009). Effect of acute exercise con cognitive control required on during an Eriksen flanker task. J. Sport Exerc. Psychol..

[67] Schmit C., Davranche K., Easthope C.S., Colson S.S., Brisswalter J., Radel R. (2015). Pushing to the limits: The dynamics of cognitive control during exhausting exercise. Neuropsychologia.

[68] Ali A., D’Donnelll J., Foskett A., Rutherfurd-Markwick K. (2016). The influence of caffeine ingestion on strength and power performance in female team-sport players. J. Int. Soc. Sports Nutr..

[69] Baker L.B., Nuccio R.P., Jeukendrup A.E. (2014). Acute effects of dietary constituents on motor skill and cognitive performance in athletes. Nutr. Rev..

[70] Bottoms L.M., Hunter A.M., Galloway S.D.R. (2006). Effects of carbohydrate ingestion in skill maintenance in squash players. Eur. J. Sport Sci..

[71] Collardeau M., Brisswalter J., Vercruyssen F., Audiffren M., Goubault C. (2001). Single and choice reaction time during prolonged exercise in trained subjects: Influence of carbohydrate avaibility. Eur. J. Appl. Physiol..

[72] Lieberman H.R., Falco C.M., Slade S.S. (2002). Carbohydrate administration during a day of sustained aerobic activity improves vigilance, as assessed by a novel ambulatory monitoring device, and mood. Am. J. Clin. Nutr..

[73] Chong E., Guelfi K.J., Fournier P.A. (2011). Effect of carbohydrate mouth rinse on maximal sprint performance in competitive male cyclists. J. Sci. Med. Sport.

[74] Jeukendrup A.E., Rollo I., Carter J.M. (2013). Carbohydrate mouth rinse: Performance effects and mechanisms. Sport Sci. Exch..

[75] Beelen M., Berghuis J., Bonaparte B., Ballak S.B., Jeukendrup A.E., van Loon L.J.C. (2009). Carbohydrate mouth rinsing in the fed state: Lack of enhancement of time-trial performance. Int. J. Sport Nutr. Exerc. Metab..

[76] Devenney S., Collins K., Shortall M. (2016). Effects of various concentrations of carbohydrate mouth rise on cycling performance in a fed state. Eur. J. Sport. Sci..

[77] Fraga C., Velasques B., Koch A.J., Machado M., Paulucio D., Ribeiro P., Pompeu F.A. (2017). Carbohydrate mouth rinse enhances time to exhaustion during treadmill exercise. Clin. Physiol. Funct. Imaging.

[78] Clarke N.D., Kornilios E., Richardson D.L. (2015). Carbohydrate and caffeine mouth rinses do not affect maximum strength and muscular endurance performance. J. Strength Cond. Res..

[79] Lane J., Steege J., Rupp S., Kuhn C. (1992). Menstrual cycle effects on caffeine elimination in the human female. Eur. J. Clin. Pharmacol..

[80] Kumar S., Mufti M., Kisan R. (2013). Variation of reaction time in different phases of menstrual cycle. J. Clin. Diagn. Res..

