# Supplementary material for: Cognitive Performance Enhancement Induced by Caffeine, Carbohydrate and Guarana Mouth Rinsing during Submaximal Exercise
Source: Nutrients. 2017 Jun 9;9(6):589. doi: 10.3390/nu9060589 (PMC5490568; doi:10.3390/nu9060589)
Supplement: Supplementary file 1 [file nutrients-09-00589-s001.zip › nutrients-194721-supplementary.pdf]

			Duration Production Task mean (standard errors)		Simon Task mean (standard errors)							
			Produced Duration (ms)	Variance (ms)	Reaction Time (ms)				Errors (%)			
					CO	IN	Total	Simon Effect §	CO	IN	Total	Simon Effect §
Nutritional supplementation	PL	Block 1	1370.7 (50.9)	197.9 (17.5)	336.0 (8.7)	365.5 (9.7)	350.7 (6.8)	29.4 (5.7)	4.7 (0.8)	9.8 (1.2)	7.2 (0.8)	5.1 (1.2)
		Block 2	1313.3 (56.8)	201.4 (24.0)	334.3 (7.3)	359.8 (9.0)	347.1 (6.0)	25.5 (5.1)	4.3 (0.9)	8.9 (1.3)	6.6 (0.9)	4.6 (1.3)
		Block 3	1312.7 (58.6)	185.6 (15.9)	323.9 (7.7)	357.6 (7.9)	340.8 (6.0)	33.7 (3.7)	4.6 (0.6)	8.7 (1.2)	6.6 (0.7)	4.2 (1.5)
		Total	1332.2 (31.8)	195.0 (11.1)	331.4 (4.6) ●	361.1 (5.1) ●	346.2 (3.6)	29.5 (2.8) ●	4.5 (0.4)	9.1 (0.7)	6.8 (0.5)	4.6 (0.8)
	CHO	Block 1	1348.9 (44.2)	186.8 (14.5)	334.7 (8.1)	362.2 (8.0)	348.4 (6)	27.5 (6.3)	7.0 (1.5)	10.4 (1.7)	8.7 (1.2)	3.4 (2.6)
		Block 2	1259.2 (41.0)	171.6 14.6)	334.4 (8.4)	361.8 (8.5)	348.1 (6.1)	27.5 (5.5)	3.3 (0.8)	9.5 (0.9)	6.4 (0.8)	6.3 (1.0)
		Block 3	1249.6 (39.4)	161.5 (11.8)	324.2 (8.0)	356.5 (8.1)	354.6 (6.1)	32.3 (5.5)	4.4 (0.9)	9.8 (1.1)	7.1 (0.8)	5.3 (1.2)
		Total	1285.9 (24.3) ◘	173.3 (7.9) ◘ ▲	331.1 (4.7) ●	360.2 (4.7)	345.6 (3.5)	29.1 (3.3) ●	4.9 (0.7)	9.9 (0.7)	7.4 (0.5)	5.0 (1.0)
	CAF	Block 1	1292.6 (24.5)	187.0 (11.8)	338.0 (8.5)	364.4 (8.9)	351.2 (6.3)	26.4 (5.12)	5.1 (0.9)	10.3 (1.3)	7.7 (0.9)	5.2 (1.6)
		Block 2	1255.9 (39.1)	187.3 (14.3)	336.8 (8.0)	360.7 (6.7)	348.8 (5.5)	23.9 (4.6)	3.9 (0.9)	10.5 (1.4)	7.2 (0.9)	6.6 (1.6)
		Block 3	1249.0 (31.5)	167.7 (11.5)	328.4 (7.4)	352.4 (7.4)	340.4 (5.5)	24 (4.7)	4.6 (0.8)	8.5 (1.4)	6.5 (0.9)	3.9 (1.7)
		Total	1265.8 (18.5) ◘	180.7 (7.3) ◘	334.4 (4.6)	359.1 (4.4)	346.8 (3.3)	24.8 (2.8)	4.5 (0.5)	9.8 (0.8)	7.1 (0.5)	5.3 (0.9)
	GUA	Block 1	1311.2 (35.4)	193.7 (15.8)	338.7 (7.9)	370.4 (8.7)	354.6 (6.3)	31.7 (6.4)	5.8 (1.1)	10.5 (1.5)	8.2 (1.0)	4.6 (1.8)
		Block 2	1249.9 (40.4)	176.5 (13.1)	336.8 (7)	365.8 (7.9)	351.3 (5.7)	29 (5.6)	3.8 (0.9)	13.3 (2.3)	8.6 (1.4)	9.5 (2.6)
		Block 3	1281.1 (40.2)	170.5 (13.2)	326.9 (7.9)	360.1 (7.6)	343.5 (6.0)	33.2 (5.4)	3.0 (0.7)	12.0 (1.6)	7.5 (1.1)	9.0 (2.8)
		Total	1280.7 (22.2) ◘	180.2 (8.1) ◘	334.1 (4.4) ■	365.4 (4.6) ●	349.8 (3.4)	30.0 (3.3) ●	4.2 (0.6)	11.9 (1.1)	8.1 (0.7)	7.7 (1.2)
Exercise		Block 1	1331.0 (20.0) $	191.3 (7.4) $	336.9 (4.1)	365.5 (4.4)	351.2 (3.2) $	28.4 (2.9)	5.7 (0.6)	10.2 (0.7)	8.0 (0.5)	8.0 (0.5)
		Block 2	1269.8 (22.3) *	184.3 (8.5) $	335.6 (3.8)	362.0 (4.0)	348.8 (2.9) $	26.2 (2.6)	3.8 (0.4)	10.5 (0.8)	7.1 (0.5)	7.1 (0.5)
		Block 3	1273.0 (21.7) *	171.3 (6.6) *	325.9 (3.8)	356.6 (3.8)	340.9 (2.9)	30.5 (2.4)	4.1 (0.4)	9 .7 (0.7)	6.8 (0.4) ~	6.8 (0.4)
		Total	1292.2 (12.4)	182.9 (4.4)	332.9 (2.4)	361.4 (2.3)	347.2 (2.0)	29.95 (1.8)	4.5 (0.3)	10.2 (0.4)	7.4 (0.3)	5.6 (0.5)

			Duration Production Task mean (standard errors)		Simon Task mean (standard errors)							
			Produced Duration (ms)	Variance (ms)	Reaction Time (ms)			Simon Effect §	Errors (%)			Simon Effect §
					CO	IN	Total		CO	IN	Total	
Nutritional supplementation	PL	Block 1	1370.7 (50.9)	197.9 (17.5)	336.0 (8.7)	365.5 (9.7)	350.7 (6.8)	29.4 (5.7)	4.7 (0.8)	9.8 (1.2)	7.2 (0.8)	5.1 (1.2)
		Block 2	1313.3 (56.8)	201.4 (24.0)	334.3 (7.3)	359.8 (9.0)	347.1 (6.0)	25.5 (5.1)	4.3 (0.9)	8.9 (1.3)	6.6 (0.9)	4.6 (1.3)
		Block 3	1312.7 (58.6)	185.6 (15.9)	323.9 (7.7)	357.6 (7.9)	340.8 (6.0)	33.7 (3.7)	4.6 (0.6)	8.7 (1.2)	6.6 (0.7)	4.2 (1.5)
		Total	1332.2 (31.8)	195.0 (11.1)	331.4 (4.6) •	361.1 (5.1) •	346.2 (3.6)	29.5 (2.8) •	4.5 (0.4)	9.1 (0.7)	6.8 (0.5)	4.6 (0.8)
	CHO	Block 1	1348.9 (44.2)	186.8 (14.5)	334.7 (8.1)	362.2 (8.0)	348.4 (6)	27.5 (6.3)	7.0 (1.5)	10.4 (1.7)	8.7 (1.2)	3.4 (2.6)
		Block 2	1259.2 (41.0)	171.6 14.6)	334.4 (8.4)	361.8 (8.5)	348.1 (6.1)	27.5 (5.5)	3.3 (0.8)	9.5 (0.9)	6.4 (0.8)	6.3 (1.0)
		Block 3	1249.6 (39.4)	161.5 (11.8)	324.2 (8.0)	356.5 (8.1)	354.6 (6.1)	32.3 (5.5)	4.4 (0.9)	9.8 (1.1)	7.1 (0.8)	5.3 (1.2)
		Total	1285.9 (24.3) ◘	173.3 (7.9) ◘ ▲	331.1 (4.7) •	360.2 (4.7)	345.6 (3.5)	29.1 (3.3) •	4.9 (0.7)	9.9 (0.7)	7.4 (0.5)	5.0 (1.0)
	CAF	Block 1	1292.6 (24.5)	187.0 (11.8)	338.0 (8.5)	364.4 (8.9)	351.2 (6.3)	26.4 (5.12)	5.1 (0.9)	10.3 (1.3)	7.7 (0.9)	5.2 (1.6)

		Block 2	1255.9 (39.1)	187.3 (14.3)	336.8 (8.0)	360.7 (6.7)	348.8 (5.5)	23.9 (4.6)	3.9 (0.9)	10.5 (1.4)	7.2 (0.9)	6.6 (1.6)
		Block 3	1249.0 (31.5)	167.7 (11.5)	328.4 (7.4)	352.4 (7.4)	340.4 (5.5)	24 (4.7)	4.6 (0.8)	8.5 (1.4)	6.5 (0.9)	3.9 (1.7)
		Total	1265.8 (18.5) ¤	180.7 (7.3) ¤	334.4 (4.6)	359.1 (4.4)	346.8 (3.3)	24.8 (2.8)	4.5 (0.5)	9.8 (0.8)	7.1 (0.5)	5.3 (0.9)
	GUA	Block 1	1311.2 (35.4)	193.7 (15.8)	338.7 (7.9)	370.4 (8.7)	354.6 (6.3)	31.7 (6.4)	5.8 (1.1)	10.5 (1.5)	8.2 (1.0)	4.6 (1.8)
		Block 2	1249.9 (40.4)	176.5 (13.1)	336.8 (7)	365.8 (7.9)	351.3 (5.7)	29 (5.6)	3.8 (0.9)	13.3 (2.3)	8.6 (1.4)	9.5 (2.6)
		Block 3	1281.1 (40.2)	170.5 (13.2)	326.9 (7.9)	360.1 (7.6)	343.5 (6.0)	33.2 (5.4)	3.0 (0.7)	12.0 (1.6)	7.5 (1.1)	9.0 (2.8)
		Total	1280.7 (22.2) ¤	180.2 (8.1) ¤	334.1 (4.4) ■	365.4 (4.6) •	349.8 (3.4)	30.0 (3.3) •	4.2 (0.6)	11.9 (1.1)	8.1 (0.7)	7.7 (1.2)
Exercise		Block 1	1331.0 (20.0) $	191.3 (7.4) $	336.9 (4.1)	365.5 (4.4)	351.2 (3.2) $	28.4 (2.9)	5.7 (0.6)	10.2 (0.7)	8.0 (0.5)	8.0 (0.5)
		Block 2	1269.8 (22.3) *	184.3 (8.5) $	335.6 (3.8)	362.0 (4.0)	348.8 (2.9) $	26.2 (2.6)	3.8 (0.4)	10.5 (0.8)	7.1 (0.5)	7.1 (0.5)
		Block 3	1273.0 (21.7) *	171.3 (6.6) *	325.9 (3.8)	356.6 (3.8)	340.9 (2.9)	30.5 (2.4)	4.1 (0.4)	9 .7 (0.7)	6.8 (0.4) ~	6.8 (0.4)
		Total	1292.2 (12.4)	182.9 (4.4)	332.9 (2.4)	361.4 (2.3)	347.2 (2.0)	29.95 (1.8)	4.5 (0.3)	10.2 (0.4)	7.4 (0.3)	5.6 (0.5)
